# Dipeptidyl Peptidase-4 (DPP-4) Inhibitors Versus Glucagon-Like Peptide-1 (GLP-1) Receptor Agonists Following Orthopedic Shoulder Surgery: A Comparative Analysis

**DOI:** 10.7759/cureus.110874

**Published:** 2026-06-15

**Authors:** Ronak J Mahatme, Shawn A Moore, Anish Gangavaram, Nishanth Muthusamy, David L Bernholt, Brian M Grawe

**Affiliations:** 1 Department of Orthopaedics, University of Cincinnati College of Medicine, Cincinnati, USA; 2 Department of Orthopaedic Surgery, University of Cincinnati College of Medicine, Cincinnati, USA

**Keywords:** dipeptidyl peptidase 4 (dpp-4), glucagon like peptide (glp-1), postoperative opioid use, shoulder surgery, type ii diabetes

## Abstract

Background: Diabetes mellitus is an independent risk factor for complications following orthopedic shoulder procedures. Dipeptidyl peptidase-4 (DPP-4) inhibitors and glucagon-like peptide-1 (GLP-1) receptor agonists are effective agents that manage type 2 diabetes, but their differential effects on perioperative and long-term shoulder outcomes remain unclear.

Methods: We conducted a retrospective cohort study using the TriNetX Research Network, identifying adults (≥18 years) with type 2 diabetes who underwent orthopedic shoulder procedures and were prescribed either a DPP-4 inhibitor or a GLP-1 receptor agonist. Propensity score matching was performed (1:1), adjusting for demographics, comorbidities, and procedure type, yielding 632 patients per cohort. Outcomes included rates of reoperation at one and two years postoperatively and postoperative opioid use.

Results: At one- and two-year follow-up, rates of reoperation were similar between cohorts. DPP-4 users had significantly higher early postoperative opioid use compared with GLP-1 users (RR: 1.754; 95% CI: 1.440-2.137; p < 0.001), whereas prolonged and chronic postoperative opioid use were similar between cohorts.

Conclusion: In patients with type 2 diabetes undergoing orthopedic shoulder procedures, DPP-4 inhibitors and GLP-1 receptor agonists demonstrated comparable long-term surgical outcomes, including reoperation rates at one and two years postoperatively. However, early postoperative opioid use was significantly higher among DPP-4 users, while prolonged and chronic opioid utilization were similar between cohorts. These findings support the perioperative safety of both incretin-based therapies and suggest that GLP-1 receptor agonists may confer additional benefit through potential central pain-modulating effects that reduce early postoperative opioid requirements.

## Introduction

Diabetes mellitus is a common systemic disease, with type 2 diabetes mellitus affecting approximately 6% of the global population [1,2]. Diabetes mellitus is an independent risk factor for numerous complications following orthopedic surgery, including infection, prolonged postoperative hospital stay, and reoperation [3,4]. These adverse outcomes likely result from the chronic inflammatory state associated with diabetes, combined with impaired circulation and reduced tissue repair, which contribute to increased complications across various orthopedic procedures [5,6].

Patients with diabetes undergoing shoulder procedures - such as arthroplasty, fracture fixation, arthroscopy, and tendon repair - often experience higher rates of complications and worse postoperative outcomes [7-9]. Specifically, diabetes has been linked to increased risks of shoulder infection, rotator cuff retear, and adhesive capsulitis [9-11]. These complications may increase healthcare utilization through subsequent reoperations and can alter pain perception, thereby affecting postoperative opioid use [12]. Accordingly, maintaining appropriate glycemic control in diabetic patients has been shown to reduce adverse postoperative complications following shoulder surgery [13,14].

Dipeptidyl peptidase-4 (DPP-4) inhibitors (e.g., sitagliptin, saxagliptin, linagliptin) and glucagon-like peptide-1 (GLP-1) receptor agonists (e.g., semaglutide, liraglutide, dulaglutide) are commonly prescribed for type 2 diabetes and act via the incretin pathway [15,16]. Despite their widespread use, few studies have examined the effects of these therapies on surgical outcomes following shoulder surgery. Furthermore, the differential impact of DPP-4 inhibitors versus GLP-1 receptor agonists on outcomes in diabetic patients, such as reoperation rates and opioid utilization, has not been thoroughly investigated.

The purpose of this study was to evaluate postoperative outcomes following orthopedic shoulder procedures in patients with type 2 diabetes treated with DPP-4 inhibitors compared with GLP-1 receptor agonists using a large national health research database. We hypothesized that outcomes would be similar between cohorts due to the shared mechanism of action via the incretin pathway.

## Materials and methods

The data used in this retrospective cohort study were collected on August 4th, 2025, from the TriNetX Research Network [17], which provided access to electronic medical records (diagnoses, procedures, medications, laboratory values, genomic information) from over 150 million patients from 102 healthcare organizations. TriNetX, LLC (Cambridge, MA, USA) [17] is compliant with the Health Insurance Portability and Accountability Act (HIPAA) and applicable data privacy regulations, and all data accessed through the platform is de-identified in accordance with federal standards. Because this study used only de-identified patient records and did not involve the collection, use, or transmittal of individually identifiable data, this study was exempted from Institutional Review Board approval.

We identified adult patients (≥18 years) with type 2 diabetes mellitus who underwent non-diagnostic shoulder arthroscopy and open procedures involving repair, reconstruction, fracture, or dislocation treatment of the shoulder from October 1st, 2003, to October 1st, 2023. The DPP-4 cohort consisted of patients prescribed a DPP-4 inhibitor within six months prior to surgery and within two years postoperatively. The GLP-1 cohort consisted of patients prescribed a GLP-1 receptor agonist during the same timeframe. Because medication administration records and perioperative withholding practices were not available within TriNetX, this study evaluated documented medication exposure rather than confirmed perioperative continuation. Therefore, we could not determine whether GLP-1 receptor agonists were held prior to surgery in accordance with evolving perioperative guidance.

Patients with polytrauma within six months of surgery were excluded, as were those prescribed both a DPP-4 inhibitor and a GLP-1 agonist during the study window in order to isolate drug-specific effects. Additional exclusions included a preoperative diagnosis of type 1 diabetes mellitus, systemic connective tissue disorders, long-term corticosteroid use, autoinflammatory syndromes, inflammatory polyarthropathies, prior stress or pathological fractures involving the shoulder or humerus, osteoporosis with current pathological fracture, or prior surgical procedures of the shoulder.

Propensity score matching was performed at a 1:1 ratio using demographic and clinical variables, including age at the time of surgery, sex, osteoporosis, body mass index, tobacco use, comorbidities, and procedure type. Following matching, both the DPP-4 and GLP-1 cohorts included 632 patients (Table 1).

**Table 1:** Propensity Score Matching for Patients With Type 2 Diabetes Mellitus Undergoing Orthopedic Shoulder Procedures Standardized mean differences (SMDs) <0.10 were considered an acceptable balance. ICD-10-CM and CPT codes used for propensity score matching are provided following each diagnosis/procedure. DPP-4: dipeptidyl peptidase-4 inhibitors; GLP-1: glucagon-like peptide-1 receptor agonists; ICD-10-CM: International Classification of Diseases, 10th Revision, Clinical Modification; CPT: Current Procedural Terminology; ORIF: open reduction and internal fixation; MDI: multidirectional instability

Characteristic	Before Matching			After Matching		
	DPP-4 (% of Cohort)	GLP-1 (% of Cohort)	SMD	DPP-4 (% of Cohort)	GLP-1 (% of Cohort)	SMD
Total	733 (100%)	1,350 (100%)	-	632 (100%)	632 (100%)	-
Demographics						
Age at index	64.0 ± 10.1 (100%)	59.1 ± 9.3 (100%)	0.5	62.8 ± 9.8 (100%)	62.6 ± 8.9 (100%)	0.025
Female	319 (43.5%)	660 (48.9%)	0.108	280 (44.3%)	290 (45.9%)	0.032
Male	404 (55.1%)	643 (47.6%)	0.15	343 (54.3%)	338 (53.5%)	0.016
Unknown sex	10 (1.4%)	47 (3.5%)	0.13	9 (1.4%)	4 (0.6%)	0.078
Diagnosis						
Osteoporosis without current pathological fracture (ICD-10-CM: M81)	44 (6.0%)	72 (5.3%)	0.029	37 (5.9%)	42 (6.6%)	0.033
BMI 30-39, adult (ICD-10-CM: Z68.3)	109 (14.9%)	376 (27.9%)	0.321	106 (16.8%)	93 (14.7%)	0.056
BMI 40+, adult (ICD-10-CM: Z68.4)	41 (5.6%)	216 (16.0%)	0.34	41 (6.5%)	35 (5.5%)	0.04
Tobacco use (ICD-10-CM: Z72.0)	21 (2.9%)	57 (4.2%)	0.073	21 (3.3%)	14 (2.2%)	0.068
Heart failure (ICD-10-CM: I50)	55 (7.5%)	109 (8.1%)	0.021	50 (7.9%)	38 (6.0%)	0.075
Chronic kidney disease (ICD-10-CM: N18)	132 (18.0%)	185 (13.3%)	0.129	102 (16.1%)	109 (17.2%)	0.03
Hypertensive diseases (ICD-10-CM: I10–I1A)	641 (87.4%)	1,174 (87.0%)	0.015	553 (87.5%)	553 (87.5%)	<0.001
Dyslipidemia (ICD-10-CM: E78)	577 (78.7%)	1,180 (87.4%)	0.233	513 (81.2%)	523 (82.8%)	0.041
Chronic ischemic heart disease (ICD-10-CM: I25)	163 (22.2%)	285 (21.1%)	0.027	141 (22.3%)	138 (21.8%)	0.011
Procedure						
Muscle transfer, shoulder/upper arm (CPT: 1004202)	10 (1.4%)	14 (1.0%)	0.061	10 (1.6%)	10 (1.6%)	<0.001
Tenotomy, shoulder (CPT: 1004206)	10 (1.4%)	23 (1.7%)	0.028	10 (1.6%)	10 (1.6%)	<0.001
Shoulder arthroscopy (CPT: 1005614)	426 (58.1%)	1,031 (76.4%)	0.396	406 (64.2%)	417 (66.0%)	0.037
Rotator cuff repair, open (CPT: 1004209)	63 (8.6%)	83 (6.1%)	0.094	50 (7.9%)	48 (7.6%)	0.012
Arthroplasty, glenohumeral (CPT: 1004224)	218 (29.7%)	201 (14.9%)	0.363	156 (24.7%)	150 (23.7%)	0.022
Prophylactic fixation (CPT: 1004229)	10 (1.4%)	0 (0%)	0.166	0 (0%)	0 (0%)	-
Osteotomy, clavicle (CPT: 1014575)	0 (0%)	10 (0.7%)	0.122	0 (0%)	0 (0%)	-
Coracoacromial ligament release (CPT: 23415)	10 (1.4%)	10 (0.7%)	0.061	10 (1.6%)	10 (1.6%)	<0.001
Rotator cuff reconstruction (CPT: 23420)	10 (1.4%)	10 (0.7%)	0.061	10 (1.6%)	10 (1.6%)	<0.001
Biceps tenodesis (CPT: 23430)	115 (15.7%)	201 (14.9%)	0.022	99 (15.7%)	104 (16.5%)	0.022
Biceps tendon resection/transplant (CPT: 23440)	10 (1.4%)	20 (1.5%)	0.01	10 (1.6%)	10 (1.6%)	<0.001
Posterior capsulorrhaphy (CPT: 23465)	10 (1.4%)	10 (0.7%)	0.061	10 (1.6%)	10 (1.6%)	<0.001
MDI capsulorrhaphy (CPT: 23466)	10 (1.4%)	0 (0%)	0.166	0 (0%)	0 (0%)	-
Proximal humerus fracture ORIF (CPT: 1014066)	46 (6.3%)	45 (3.3%)	0.138	30 (4.7%)	36 (5.7%)	0.043
Acromioclavicular dislocation repair (CPT: 1014577)	10 (1.4%)	10 (0.7%)	0.061	10 (1.6%)	10 (1.6%)	<0.001
Clavicle fracture ORIF (CPT: 23515)	10 (1.4%)	10 (0.7%)	0.061	10 (1.6%)	10 (1.6%)	<0.001
Scapular fracture ORIF (CPT: 23585)	10 (1.4%)	10 (0.7%)	0.061	10 (1.6%)	10 (1.6%)	<0.001
Greater tuberosity ORIF (CPT: 23630)	10 (1.4%)	10 (0.7%)	0.061	10 (1.6%)	10 (1.6%)	<0.001
Shoulder dislocation ORIF (CPT: 23660)	0 (0%)	10 (0.7%)	0.122	0 (0%)	0 (0%)	-

Rates of shoulder reoperation at one and two years postoperatively were assessed. Additionally, postoperative opioid use was categorized into early (1-30 days), prolonged (30-90 days), and chronic (90-180 days) postoperative exposure windows. Medication utilization reflected recorded prescription activity within each interval and did not incorporate cumulative morphine milligram equivalent thresholds or confirm medication consumption.

Patient queries, demographic factors, and outcome of interest were identified using Current Procedural Terminology (CPT; a standardized procedural coding system maintained by the American Medical Association), International Classification of Diseases, 10th Revision, Clinical Modification (ICD-10-CM; a standardized diagnostic coding system maintained by the Centers for Disease Control and Prevention), and RxNorm (a standardized medication nomenclature developed by the National Library of Medicine) codes [18-20]. All codes used are provided in the Appendices.

Risk ratios (RRs) and 95% confidence intervals (CIs) were computed, and the complication rates were analyzed using the TriNetX system [17]. Categorical variables were assessed using the chi-squared test, while continuous variables were evaluated with independent t-tests. The level of statistical significance was set at P < 0.05.

## Results

Rates of reoperation at both one year (RR: 1.182; 95% CI: 0.533-2.618; p = 0.837) and two years (RR: 0.667; 95% CI: 0.371-1.198; p = 0.225) postoperatively were similar between patients on DPP-4 and GLP-1 (Table 2).

**Table 2:** Postoperative Opioid Use and Reoperation Outcomes Between DPP-4 (n = 632) and GLP-1 (n = 632) Cohorts Following Shoulder Surgery Analyses were performed on a 1:1 propensity-score-matched cohort. Categorical outcomes were compared using Pearson chi-square tests (χ^2^). Risk ratios were calculated using the GLP-1 cohort as the reference group. CI: confidence interval; DPP-4: dipeptidyl peptidase-4 inhibitors; GLP-1: glucagon-like peptide-1 receptor agonists

Outcome	DPP-4 Events (%)	GLP-1 Events (%)	Risk Ratio	95% CI	χ^2^ Statistic	P-value
Early postoperative opioid use	207 (32.8)	118 (18.7)	1.754	1.440-2.137	32.075	<0.001
Prolonged postoperative opioid use	123 (19.5)	104 (16.5)	1.183	0.933-1.499	1.740	0.187
Chronic postoperative opioid use	120 (19.0)	109 (17.2)	1.101	0.871-1.392	0.533	0.465
One-year reoperation	13 (2.1)	11 (1.7)	1.182	0.533-2.618	0.042	0.837
Two-year reoperation	18 (2.8)	27 (4.3)	0.667	0.371-1.198	1.475	0.225

The DPP-4 cohort had significantly greater early postoperative opioid use compared to the GLP-1 cohort (RR: 1.754; 95% CI: 1.440-2.137; p < 0.001). There were no significant differences in prolonged postoperative opioid use (RR: 1.183; 95% CI: 0.933-1.499; p = 0.187) and chronic postoperative opioid use (RR: 1.101; 95% CI: 0.871-1.392; p = 0.465).

## Discussion

To the best of our knowledge, this study represents the first comparative analysis examining postoperative outcomes in patients with type 2 diabetes prescribed DPP-4 inhibitors versus those prescribed GLP-1 receptor agonists undergoing orthopedic shoulder procedures. Our findings extend prior metabolic and cardiovascular research on incretin-based therapies to the musculoskeletal surgical domain, demonstrating that while long-term rates of reoperation were similar between cohorts, patients using DPP-4 inhibitors exhibited significantly higher rates of early postoperative opioid use [15,16]. Notably, this difference did not persist, as opioid utilization was comparable between groups during the prolonged and chronic postoperative periods.

Reoperation rates at both one- and two-year follow-up were comparable between the DPP-4 and GLP-1 cohorts, suggesting that these medications do not differentially affect perioperative healing or the likelihood of requiring additional surgery. While GLP-1 receptor agonists are generally more effective than DPP-4 inhibitors in reducing glycated hemoglobin, promoting weight loss, and improving cardiovascular outcomes, DPP-4 inhibitors also achieve sufficient glycemic control to mitigate diabetes-related impairments in tissue repair and postoperative recovery [7-9,15,16,21,22]. These findings align with prior evidence indicating that improved glycemic control, whether through DPP-4 or GLP-1-based therapy, reduces tendon retear risk following arthroscopic rotator cuff repair [13]. Moreover, Khan et al. reported that elevated glycated hemoglobin was not associated with increased reoperation risk after rotator cuff repair in diabetic patients, further supporting our observation of similar long-term outcomes between cohorts [23]. Although both incretin-based therapies may positively influence bone mineral density, there remains no direct evidence linking these mechanisms to orthopedic healing, which is consistent with our findings of comparable reoperation rates [24,25].

The observed difference in early postoperative opioid use may reflect differences in pain modulation, glycemic control, or other unmeasured perioperative factors rather than differences in structural recovery. Diabetes has been associated with increased postoperative opioid requirements, and prior preclinical and clinical studies have suggested that GLP-1 receptor agonists may influence pain signaling pathways [26-32]. Proposed mechanisms include modulation of central gamma-aminobutyric acid (GABA) signaling and attenuation of neuropathic pain responses [27-32]. However, because pain scores and medication adherence were unavailable, the present findings should be interpreted cautiously and should not be interpreted as evidence of a direct analgesic effect of GLP-1 therapy.

This study has several limitations. The use of a claims-based database introduces potential coding inaccuracies and exposure misclassification. Although propensity matching controlled for numerous demographic and clinical variables, residual confounding remains possible, including factors such as diabetes severity, glycated hemoglobin levels, peripheral neuropathy, and differences in perioperative care pathways. Medication exposure was determined from prescription records and did not confirm medication adherence, continuous use, or whether GLP-1 receptor agonists were withheld perioperatively according to evolving institutional protocols. Variability in postoperative rehabilitation, opioid prescribing practices, and perioperative pain management across institutions could not be standardized. Additionally, opioid utilization reflected documented prescription activity rather than confirmed consumption or cumulative morphine equivalent dosing. Patient-reported outcomes and functional recovery measures were unavailable. Finally, although procedure type was included in propensity matching, residual heterogeneity in operative complexity may remain across shoulder procedures. Despite these limitations, this study provides useful observational evidence from a large propensity score-matched multicenter cohort of patients with type 2 diabetes undergoing shoulder surgery. Although causality cannot be established, the absence of differences in reoperation outcomes and the isolated association with early postoperative opioid utilization suggest that major differences in measured postoperative outcomes were not observed within this matched population. These findings provide preliminary context for perioperative decision-making and support future investigation into medication-specific effects on postoperative pain and recovery.

## Conclusions

In patients with type 2 diabetes undergoing orthopedic shoulder procedures, DPP-4 inhibitors and GLP-1 receptor agonists demonstrated comparable long-term surgical outcomes, including reoperation rates at one and two years postoperatively. However, early postoperative opioid use was significantly higher among DPP-4 users, while prolonged and chronic opioid utilization were similar between cohorts. These findings suggest comparable short-term and intermediate postoperative outcomes between incretin-based therapies in this matched cohort and support future investigation into factors associated with postoperative opioid utilization.

## Appendices

**Table 3:** CPT, ICD-10-CM, and RxNorm Codes for TrinetX Queries, Inclusions, Exclusions, Propensity Score Matching, and Outcomes DPP-4: dipeptidyl peptidase-4 inhibitors; GLP-1: glucagon-like peptide-1 receptor agonists; ICD-10-CM: International Classification of Diseases, 10th Revision, Clinical Modification; CPT: Current Procedural Terminology

Diagnosis	Codes
Orthopedic Shoulder Surgery	1004202, 1004206, 1004209, 1004216, 1004219, 1004224, 1004229, 1014575, 23400, 23415, 23420, 23420, 23440, 23465, 23466, 1014066, 1014576, 1014577, 23515, 23585, 23630, 23660, 23670, 23680, 1005614
Type 2 Diabetes Mellitus	E11
DPP-4 Inhibitors	593411, 857974, 1100699, 1368001
GLP-1 Receptor Agonists	60548, 1440051, 60548, 475968, 1551291, 1991302, 1534763
Tirzepatide (excluded)	2601723
Polytrauma	T07
Exclusion Criteria (type 1 diabetes mellitus, systemic connective tissue disorders, long-term corticosteroid use, autoinflammatory syndromes, inflammatory polyarthropathies, prior stress or pathological fractures involving the shoulder or humerus, osteoporosis with current pathological fracture, or prior surgical procedures on the shoulder)	E10, M30-M36, Z79.5, Q79.6, Q87.4, M04, M05-M14, M84.31, M84.32, M84.41, M84.42, M84.51, M84.52, M84.61, M84.62, M80, 1004147, 1005614, 29805
Propensity Score Inputs	Age at Index, Female, Male, M81, Z68.3, Z68.4, Z72.0, I50, N18, I10-I1A, E78, I25, 1004202, 1004206, 1004209, 1004216, 1004219, 1004224, 1004229, 1014575, 23400, 23415, 23420, 23430, 23440, 23465, 23466, 1014066, 1014576, 1014577, 23515, 23585, 23630, 23660, 23670, 23680, 1005614
Reoperation	1004147, 1005614, 29805
Opioid Use	5489, 7052, 3423, 7804, 10689
